# Concomitant Trajectories of Internalising, Externalising, and Peer Problems Across Childhood: a Person-centered Approach

**DOI:** 10.1007/s10802-021-00851-8

**Published:** 2021-07-19

**Authors:** Lisa-Christine Girard

**Affiliations:** School of Health in Social Science, Clinical Psychology, University of Edinburgh, South Bridge, Edinburg, EH8 9YL UK

**Keywords:** Internalising problems, Externalising problems, Peer problems, Multi-group-based trajectories, Cohort study, Childhood

## Abstract

This study investigated trajectories of concomitant internalising, externalising, and peer problems, and associated risk factors for group-membership, using a person-centered approach to better understand heterogeneity in subgroups identified. A cohort of 7,507 children in Ireland was followed from infancy to late childhood (50.3%, males; 84.9% Irish). The parent-version of the Strengths and Difficulties Questionnaire was used when children were 3, 5, 7 and 9 years of age. Information on antecedent risk factors was collected when children were 9 months. Group-based multi-trajectory modelling and multinomial logistic regression were used. Six subgroups of children with distinct profiles were identified, evidencing both homotypic and heterotypic comorbidity. No support of a ‘pure’ internalising, externalising or peer problems group was found in any identified trajectory group. Difficulties in one problem domain indicated the presence of difficulty in another problem domain for all children in elevated groups. Risk factors associated with group-membership were complex, with only three common factors across elevated groups: prenatal exposure to smoking, maternal education, and maternal stress. Specific risk factors for group-membership included low birth weight, sex, maternal age, maternal depression, family composition, social class, medical card status and quality of attachment. Despite some overlap in predictors, the combination of predictors specific to each group would suggest tailored programming. For children with the most acute problems, programming targets should include families with boys, born with low birth weight, exposed to smoking prenatally, with mothers who have lower levels of education, postnatal depression, increased stress and fewer financial resources.

**Supplementary Information:**

The online version contains supplementary material available at 10.1007/s10802-021-00851-8.

It is well understood that early emotional and behavioural pathologies are indicative of a wide range of maladaptive outcomes. For example, early internalising problems such as emotional problems and anxious/withdrawn behaviours have been commonly associated with future major depressive disorder, social and specific phobias, body dissatisfaction, alcohol-related problems and suicidality, amongst others (e.g., Caspi et al., 1996; Goodwin et al., 2004; Orri et al., 2018; Patalay et al., 2015). Early externalising problems (e.g., conduct problems, hyperactivity) have also been associated with subsequent depressive disorder and alcohol dependence, along with higher rates of school dropout, unemployment, homelessness, teenage parenthood, relationship problems, poor health, criminality, and substance abuse (e.g., Bevilacqua et al., 2018; Collishaw et al., 2004; Colman et al., 2009; Shaw et al., 2012). Moreover, early peer problems have been associated with a wealth of similar negative outcomes, including but not limited to, lower academic achievement, higher rates of truancy and school drop-out, subsequent relationship problems, externalising, and internalising problems (Woodward & Fergusson, 2000: Laird et al., 2001; Reijntjes et al., 2010). Given this vast range of maladaptive outcomes, it is important to better understand potential heterogeneity within longitudinal presentations of these difficulties across childhood. More specifically, whether there are subgroups of children following distinct trajectories of concomitant internalising, externalising, and peer problems, as the pathways to poorer outcomes, along with their etiology, may differ across distinct groups. In the current study, concomitant trajectories of internalising (i.e., emotional problems), two externalising (i.e., conduct problems and hyperactivity/inattention), and peer problems were modelled from early to late childhood.

Developmental theories, such as the early childhood perspective of aggression (Tremblay et al., 2018) and the dual taxonomy of antisocial behaviour (Moffitt, 1993), have shown that children with the most elevated levels of early and sustained behavioural problems for example, are those with the poorest outcomes in adulthood, particularly if no remediation or interventions are sought. However, developmental research suggests that for a large proportion of children, early displays of externalising behaviours such as aggression and conduct problems are normative in the toddler years and typically decline for the majority around the time when children enter formal schooling, when higher-order skills (e.g., language, impulse control, emotional regulation) are better developed (Cole et al., 2011; Tremblay, 2010; Tremblay et al., 2004). Only a small percentage of children, between 5–10%, within population-based studies (e.g., Girard et al., 2019; Nagin & Tremblay, 1999; Odgers et al., 2007; Shaw et al., 2003, 2005) have been found to continue with elevated chronic levels of aggression and conduct problems into and across adolescence. Population-based studies have suggested higher levels, between ~ 15–20% of children, with elevated chronic levels of hyperactivity/inattention from childhood to adolescence (e.g., Galéra et al., 2011, 2021; Pingault et al., 2013; Shaw et al., 2005), with up to 40% having continuing and elevated levels into adulthood (e.g., Daley, 2006). On the other hand, internalising problems (e.g., emotional problems, anxious/depressed and withdrawn) are more likely to increase from early childhood onwards, particularly for girls (e.g., Gilliom & Shaw, 2004; Leve et al., 2005). Similar mechanisms (i.e., higher-order skill development such as cognitive and emotional regulation) have been suggested for increases found in internalising problems across childhood and adolescence, given that these skills bring with them a better capacity for reflection, rumination, and self-blame (e.g., Garnefski et al., 2005). Population-based studies have found that between 5–15% of children experience high stable and/or high rising trajectories of internalising problems across childhood/adolescence, and between 30–55% experience moderate stable or moderate rising trajectories (e.g., Veldman et al., 2015; Côté et al., 2009; Toumbourou et al., 2011). Studies examining developmental trajectories of peer problems have suggested between 10–25% of children are experiencing increasing trajectories of peer victimisation, with close to 5% of children experiencing chronically high levels across time (e.g., Barker et al., 2008; Boivin et al., 2010). This is of particular concern as taken together, up to 30% of children may be experiencing high increasing and/or chronic levels of peer problems starting in early childhood. Given the bidirectional association between peer problems and both internalising and externalising problems across childhood and adolescence (e.g., Vaillancourt et al., 2013), there is a strong need to simultaneously examine developmental trajectories of internalising, externalising, and peer problems starting in early childhood, to better understand potential sensitive periods when comorbidity or multimorbidity are most likely to emerge.

## Comorbidity

Comorbidity, (and by extension multimorbidity), a common occurrence with internalising, externalising, and peer problems, places children at even greater risk for subsequent maladaptive outcomes (Newman et al., 1998). Studies of comorbidity have found higher odds ratios for concurrent homotypic comorbidity (i.e., comorbidity within behavioural domains such as conduct disorder and hyperactivity), as compared to concurrent heterotypic comorbidity (i.e., across domains – internalising and externalising). However, certain heterotypic comorbidity (e.g., conduct disorder and depression) has been found to present with similarly high odds (Angold et al., 1999), and particularly when the comorbidity is successive. Thus, consideration of comorbidity (and multimorbidity where possible), should also be of high priority when examining developmental models of psychopathology. Yet few studies to date have modelled concomitant trajectories of both internalising and externalising problems, with some exceptions (e.g., Nivard et al., 2017; Murray et al., 2020; Patalay et al., 2017), in particular starting as early as late toddlerhood (e.g., Fanti & Henrich, 2010), whilst using person-centered approaches. Moreover, no study to the best of my knowledge, has also included peer problems when modelling concomitant internalising and externalising trajectories starting in early childhood.

### Joint and Multi-trajectories of Internalising and Externalising Problems

Nivard et al. (2017) modelled internalising (major depression, generalised anxiety disorder, specific and social phobia) and externalising (oppositional defiance disorder, conduct disorder and ADHD) problems in a population-based cohort from the UK, from seven to 15 years of age. Trajectories of internalising and externalising problems were first modelled individually and then estimates of conditional probabilities were used to examine membership in internalising groups based on membership in externalising groups and vice versa. A five-group model was identified and largely suggested associated internalising and externalising trajectories (e.g., decreasing internalising membership was associated with decreasing externalising membership, increasing internalising membership was associated with increasing externalising membership). Only one group, the adolescent onset of internalising problems group, was found to be independent of having any externalising problems, supporting a ‘pure’ internalising group in this study. Murray et al. (2020), examined multi-trajectories of internalising (anxiety, depression), externalising problems (oppositional defiance disorder, conduct disorder, aggression), and ADHD in the same age range (i.e., seven to 15 years), using a cohort from Switzerland. They used the multi group-based trajectory approach, which allows for the modelling of multiple behaviours simultaneously. Six distinct groups were identified, with a majority of groups similarly displaying comorbidity across domains. That is, two groups showed low to non-existent problems across domains, one group had low initial levels of internalising, externalising and ADHD but increased across all domains across time, one group had higher initial levels of internalising, externalising and ADHD but decreased across all domains across time, and one group had chronically elevated ADHD and internalising with decreasing externalising problems. Only one group evidenced a ‘pure’ trajectory of internalising problems, similarly to Nivard et al. (2017).

A further two studies examined joint trajectories starting in earlier ages (Fanti & Henrich, 2010; Patalay et al., 2017). Fanti and Henrich (2010), examined joint trajectories of internalising and externalising problems from two to 12 years old in a US cohort. Similar to Nivard et al. (2017), individual trajectories of internalising and externalising problems were modelled first, followed by joint conditional probabilities of internalising and externalising problems. Eleven joint trajectory groups best fit the data and revealed groups with no-low problems, co-occurring internalising and externalising problems, high internalising and high decreasing externalising problems, and ‘pure’ internalising problems; similar to results found in studies with older children. Conversely, with this younger sample, evidence of ‘pure’ externalising trajectories, both moderate and chronic, were also identified. Again using a younger sample, Patalay et al. (2017), examined joint trajectories of internalising (emotional symptoms) and externalising problems (conduct problems), in a UK population-based cohort from three to 11 years. Five trajectory groups were identified with similar patterns (albeit with fewer groups) to Fanti and Henrich (2010), including support for a ‘pure’ moderate externalising trajectory group. Taken together, these studies suggest comorbidity is common when modelling joint trajectories of internalising and externalising problems in childhood and adolescence. ‘Pure’ internalising trajectories are also consistently identified across childhood and adolescence, whereas in contrast, support for ‘pure’ externalising trajectories appear to only be found in studies modelling trajectories starting from earlier ages.

### Person-Centered Approaches

Within a developmental psychopathology framework, it is understood that not all children with emotional or behavioural pathologies will present with comorbidity or even on the same pathways to poorer outcomes, as the above studies demonstrate, and so it is important to better understand individual variation in the presentation of longitudinal (and potentially comorbid/multimorbid) emotional and behavioural problems. The commonly used variable-centered approaches to the study of developmental emotional and behavioural problems have greatly advanced our knowledge, particularly regarding a better understanding of *normative* developmental change (Laursen & Hoff, 2006) and factors implicated in variation around mean-level trends in behaviour (Nagin, 2005). Despite this, the common assumptions of homogeneity and linearity in these models (Bergman et al., 2006) have their limitations as they suggest that the developmental course of behaviour *x*, will either increase or decrease over time, and in a similar fashion for the majority of individuals. We know however, that internalising, externalising, and peer problems are non-normative rather than normative behaviours, and consequently likely to have more nuanced patterns of continuity and change over time, for different clusters of individuals within the population. Thus, applying assumptions of heterogeneity and non-linearity when modelling trajectories of concomitant internalising, externalising, and peer problems (i.e., a person-centered approach), offers a complimentary view to understanding any individual patterns of change in behaviours across development.

### Risk Factors

In line with the assumption of heterogeneity when modelling concomitant trajectories of internalising, externalising, and peer problems, this assumption is carried forward to the question of whether there are differences in etiology for the subgroups of children identified. In particular, the question of ‘common’ versus ‘specific’ risk factors in the etiology of trajectories (e.g., pure, homotypic, heterotypic comorbid subgroups) needs investigating (Cohen et al., 1990). Through the use of longitudinal cohorts, it has become better understood that there is intergenerational continuity of early and sustained emotional and behavioural problems, resulting from two paths of transmission (i.e., genetic and environmental; Kim et al., 2009; Capaldi et al., 2017). Thus, the early identification of subgroups of children with emotional, behavioural, and peer problems, along with the identification of early risk factors for specific group-membership is of high importance to ‘breaking the cycle’. Some commonly examined environmental factors previously associated with internalising and externalising problems include pre- and postnatal risks (e.g., maternal prenatal smoking, premature delivery), parenting characteristics and behaviours (e.g., maternal depression, young mothers, harsh parenting) and indicators of socioeconomic status (Button et al., 2005; Spittle et al., 2009; Goodman et al., 2011; Girard et al., 2014; Girard et al., 2016; Fergusson & Lynskey, 1993).

In the current study, 13 factors were investigated and included children’s sex, preterm birth, low birth weight, stay in the neonatal intensive care unit, prenatal exposure to smoking, maternal age, maternal education, maternal depression, marital status, indicators of SES, quality of parent–child attachment, and maternal stress. While these factors are not exhaustive, they cover risk and protective factors from multiple levels of the ecological systems theory of development (Bronfenbrenner, 1994). Given the novel approach of modelling concurrent internalising, externalising, and peer problems using a person-centred approach across almost a decade, starting in very early childhood, it was important to identify whether these previously established risk factors would 1) predict group membership and 2) reveal potential heterogeneity in etiology across groups.

### Aims & Hypotheses

More valid classification of subgroups of children following pure and/or comorbid trajectories of internalising, externalising, and peer problems is needed to further our understanding of non-normative emotional and behavioural development from early to late childhood. The aims of this study are twofold. First, to examine trajectories of concomitant internalising, externalising, and peer problems from early to late childhood using a person-centered approach, to better understand prevalence and subgroups with possible concurrent and/or successive homotypic and/or heterotypic comorbidity. Second, to identify ‘common’ versus ‘specific’ risk factors for group-membership.

Grounded in the few emerging studies modelling comorbidity of internalising and externalising behavioural trajectories, it was hypothesised that a 6-group model would best fit the data. For example, it was expected that a low to non-engagers group would be identified. Both a comorbid increasing group and a comorbid decreasing group would be identified. A normative comorbid group (i.e., early moderate but decreasing externalising problems and low early but moderately increasing internalising problems) would be identified. And finally, two groups, an elevated chronic ‘pure’ externalising group and a ‘pure’ increasing internalising group, would be identified (Fanti & Henrich, 2010; Murray et al., 2020; Nivard et al., 2017; Patalay et al., 2017). Moreover, it was expected that some common risk factors would emerge across groups with higher behavioural problems (e.g., being male, exposure to maternal depression, lower maternal education, poorer quality of attachment), although it was similarly expected that the combination of risk factors across groups would likely vary. As a result, no specific hypotheses were made regarding risk or protective factors associated with differing trajectory group membership.

## Methods

Participants included children and their families enrolled in the Growing Up in Ireland (GUI) Infant cohort study, identified from the Child Benefit Register. Infants born between December 2007 and May 2008 were randomly selected to participate, with a recruitment response rate of 65% (*N* = 11,134). Further details of recruitment and study design can be found in Williams et al. (2010). Data from waves 1 to 5 were used, when children were nine months, three, five, seven and nine years old. Attrition and non-response across waves resulted in 9,789 participants at the age of three having data on the main outcomes (i.e., internalising, externalising and peer problems), 8,998 participants at the age of five, 5,281 participants at the age of seven/eight, and 8,022 participants at the age of nine. Attrition and item non-response revealed an underrepresentation of young mothers and families experiencing greater social disadvantage (e.g., lower levels of education, income, social class, lone parent families). Sampling weights were thus used, bringing the distribution of characteristics of the sample still participating at nine years, and across at least 4 waves, to within 0.5% of the population distribution (for a detailed description of the calculated sampling weights, see Quail et al., 2019). The final sample used in the current study includes 7,507 families who participated in at least 4 of the 5 waves, using the weighting factor to ensure generalizability to the population. Demographic characteristics of participants can be found in Table 1.

**Table 1:** Demographic Characteristics

	9 months
Males	3,779 (50.3%) [n = 7,507]
Born preterm	449 (6.0%) [n = 7,487]
Low birth weight	399 (5.4%) [n = 7,420]
NICU stay	1,030 (13.7%) [n = 7,502]
Ethnicity:	[n = 7,488]
Irish	6,345 (84.9%)
Other White background	772 (10.3%)
African or any other Black background	170 (2.3%)
Chinese or any other Asian background	163 (2.2%)
Other including mixed background	29 (0.4%)
Social Class:	[n = 7,507]
Professional/managerial	4,063 (54.1%)
Other non-manual/skilled manual	2,222 (29.6%)
Semi-skilled/unskilled manual	609 (8.1%)
All other gainfully occupied/unknown	25 (0.3%)
Never worked/no class	588 (7.8%)
Single parent	664 (8.9%) [n = 7,507]
Medical card status:	[n = 7,504]
Free medical card (full coverage)	1,597 (21.3%)
General practitioner card (partial coverage)	212 (2.8%)
No medical card	5,695 (75.9%)
Exposure to household smoking	2,237 (30.4%) [n = 7,366]
Maternal age less than 21 years	232 (3.1%) [n = 7,507]
Maternal education:	[n = 7,503]
Primary level/no education	119 (1.6%)
Second level	2,882 (38.4%)
Third level	4,502 (60.0%)
Maternal postnatal depression	280 (3.8%) [n = 7,417]
Means (SD)	
Quality of attachment	42.6 (2.6) [n = 7,492]
Maternal stress	31.8 (6.7) [n = 7,458]

Medical card is means-tested and issued on the basis of financial need by health services. There are 2 tiers: “full coverage,” including visits to general practitioners and prescriptions and “general practitioner only,” excluding prescriptions. For maternal education, “primary level/no education” is roughly equivalent to elementary or middle school education in the US; “second level” is roughly equivalent to having graduated high school or having a technical trade/vocational diploma in the US; and “third level” is equivalent to higher education (i.e., college or bachelor’s degree, graduate degree, or doctorate)

### Internalising/Externalising/Peer Problems

Children’s internalising, externalising, and peer problems were assessed using the parent version of the Strengths and Difficulties Questionnaire (SDQ; Goodman, 1997), when children were three, five, seven, and nine years of age. The SDQ is a behavioural screening tool assessing mental health in children ages three to 16 years, and comprises five subscales including emotional symptoms, conduct problems, hyperactivity/inattention, peer problems, and prosocial behaviours. Each subscale includes five items, rated on a 3-point scale from 0 (*not true*) to 2 (*certainly true*). Subscale scores range from 0 to 10. This study uses the emotional symptoms, conduct problems, hyperactivity/inattention, and peer problems subscales. Cronbach’s alphas for the entire cohort were as follows: emotional symptoms = 0.58 – 0.69, conduct problems = 0.56 – 0.59, hyperactivity/inattention = 0.75 – 0.80, and peer problems = 0.58 – 0.60. The SDQ has been previously well validated in the literature (e.g., Goodman, 2001). Using previously suggested cut-offs, scores on each subscale were categorised as follows: emotional symptoms were categorised as low (0–3), moderate (4), and high (5–10), conduct problems were categorised as low (0–2), moderate (3), and high (4–10), hyperactivity/inattention was categorised as low (0–4), moderate (5–6), and high (7–10), and peer problems scores were categorised as low (0–1), moderate (2), and high (3–10), (YouthInMind, 2013).

### Risk Factors

Understanding specific antecedent risk and protective factors associated with group membership at the individual and family level resulted in the inclusion of 13 possible factors, assessed at wave 1, when children were nine months. At the individual level, children’s sex (boy/girl), preterm birth derived from gestational age in weeks (delivered prior to the 37^th^ week, no/yes), low birth weight (≤ 2500 g, no/yes), and having stayed in the neonatal intensive care unit (no/yes) were assessed. Family level factors included: number of members in the household who smoked during the pregnancy (none/ ≥ 1), maternal age (≤ 21 years of age, no/yes), highest level of maternal education (primary level/no education, second level, third level), maternal depression (a score of ≥ 11 on the Centre for Epidemiological Studies Depression Scale, 8-item short version, no/yes), medical card status (free medical care, free general practitioner, no free medical care), having a partner in the home (no/yes), social class (professional/managerial, other non-manual/skilled manual, semi-skilled/unskilled manual, all other gainfully occupied/unknown, never worked/no class), quality of parent–child attachment (Maternal Postnatal Attachment Scale; Condon & Corkindale, 1998), and total maternal stress (Parental Stress Scale; Berry & Jones, 1995). The quality of attachment subscale from the Maternal Postnatal Attachment Scale consists of 9 items measuring mothers’ feelings about themselves as parents (e.g., patience and affection), along with their feelings towards their infant. Scores on items were rescaled between 1 and 5 for a possible scale range from 9 to 45. Total maternal stress was assessed using the Parental Stress Scale, which contains 18 items on a 5-point scale to assess emotional benefits, self-enrichment, personal development, demands on resources and opportunity costs, and restrictions resulting from parenthood. Possible scores range from 18–90 with higher scores indicative of higher levels of stress. Cronbach’s alpha for the entire cohort were 0.87, 0.52, and 0.74, for maternal depression, quality of attachment, and total maternal stress, respectively.

### Statistical Analysis

To examine trajectories of children’s internalising, externalising, and peer problems, group-based trajectory modelling was used. Group-based models use finite mixture models. They are a semi-parametric, person-centered approach to modelling heterogeneity of identified groups within the examined population (Nagin, 2005). A multi group-based trajectory, an extension of the group-based trajectory, was used. This extension allows the conjoint modelling of multiple behaviours simultaneously, generating a better understanding of subgroups with possible pure, homotypic or heterotypic comorbid trajectories. An overall profile of children’s behavioural problems is thus created.

A quasi-Newton procedure and censored-normal models were used to estimate parameters in the multi-trajectory group model. Model fit was subsequently evaluated using the Bayesian Information Criteria (BIC) and the Akaike Information Criterion (AIC), whereby larger more positive values represent a better model fit (Nagin, 2005; Kass & Raferty, 1995; Raferty, 1995; Schwarz, 1978), along with the average posterior probabilities of group membership by trajectory group, AvePP, (i.e., greater than 70; Nagin, 2005), which are presented in Tables 2 and 3, respectively. The AvePP is indicative of the probability that a specific participant belongs to the model’s *J* trajectory group. Evaluation of these three criteria revealed the six-group model as the best fit to this data. Nagin (2005) further suggests that assessment of the selected model include evaluation of the odds of correct classification (OCC), which represents the classification of a specific participant as X times better in the specified *j* trajectory group than by chance alone. The threshold suggested for the OCC is greater than 5, which further supported the six-group model (Table 3). Theoretical likelihood is also considered when comparing model fit. Parameter estimates of the final multi-trajectories are presented in Table 4.

**Table 2:** Comparison of the Bayesian Information Criteria (BIC) for Assessment of Model Fit

	BIC	BIC	AIC
2-group	-193,147.72 (N = 108,983)	-193,108.92 (N = 7507)	-193,008.53
3-group	-191,118.02 (N = 108,983)	-191,061.83 (N = 7507)	-190,916.44
4-group	-190,178.37 (N = 108,983)	-190,104.80 (N = 7507)	-189,914.40
5-group	-189,534.04 (N = 108,983)	-189,443.08 (N = 7507)	-189,207.68
6-group	-189,030.42 (N = 108,983)	-188,922.07 (N = 7507)	-188,641.67
7-group	-188,788.39 (N = 108,983)	-188,662.65 (N = 7507)	-188,337.24

A larger (more positive) BIC and AIC is indicative of better model fit. The smaller N BIC represents the actual sample size used in the trajectories, the larger N BIC represents the total number of assessments used within the estimation of the model across time and participants. The two presented BIC scores bracket the theoretically correct BIC score (Nagin, 2005)

**Table 3:** Model Fit Criterion of Internalising and Externalising Multi-trajectories

Trajectory Group	*n*	Average Posterior Probability of Group Membership	Odds of Correct Classification
1	1995	85.9	16.9
2	1550	77.1	13
3	2190	79.9	9.6
4	973	81.4	29.5
5	575	81.9	54.4
6	224	89.7	281.7

Average Posterior Probability of Group Membership (i.e., the probability that a specific participant belongs to the model’s *J* trajectory group) greater than 70 and an OCC (i.e., the model classifies a specific participant X times better in the specified *J* trajectory group than by chance alone) greater than 5 represents good model fit

**Table 4:** Multi-Trajectory Parameter Estimates for the 6-Group Model

Group	Parameter	Estimate	SE	*T*	*p*
Emotional Problems					
1	Intercept	-0.28	0.10	-2.67	0.008
2	Intercept	0.99	0.11	8.88	0.000
	Linear	0.18	0.02	8.55	0.000
3	Intercept	1.33	0.23	5.76	0.000
	Linear	-0.35	0.09	-3.75	0.000
	Quadratic	0.03	0.01	4.37	0.000
4	Intercept	1.44	0.16	8.98	0.000
	Linear	0.32	0.04	9.03	0.000
5	Intercept	0.23	0.22	1.04	0.298
	Linear	0.23	0.04	5.61	0.000
6	Intercept	-1.50	0.69	-2.18	0.029
	Linear	1.45	0.26	5.58	0.000
	Quadratic	-0.08	0.02	-3.49	0.001
Conduct Problems					
1	Intercept	2.61	0.24	10.98	0.000
	Linear	-0.83	0.09	-9.28	0.000
	Quadratic	0.05	0.01	6.22	0.000
2	Intercept	2.72	0.28	9.58	0.000
	Linear	-0.59	0.10	-5.90	0.000
	Quadratic	0.03	0.01	3.80	0.001
3	Intercept	3.81	0.25	15.46	0.000
	Linear	-0.60	0.08	-7.96	0.000
	Quadratic	0.03	0.01	5.02	0.000
4	Intercept	5.26	0.39	13.52	0.000
	Linear	-0.78	0.13	-6.18	0.000
	Quadratic	0.04	0.01	4.45	0.000
5	Intercept	3.74	0.29	13.06	0.000
	Linear	-0.17	0.03	-5.23	0.000
6	Intercept	3.82	0.21	17.87	0.000
Hyperactivity/Inattention					
1	Intercept	2.07	0.11	19.12	0.000
	Linear	-0.16	0.02	-9.44	0.000
2	Intercept	1.99	0.14	14.22	0.000
3	Intercept	3.50	0.19	18.76	0.000
4	Intercept	4.22	0.15	28.49	0.000
5	Intercept	2.08	0.56	3.72	0.000
	Linear	1.39	0.21	6.61	0.000
	Quadratic	-0.09	0.02	-5.73	0.000
6	Intercept	5.04	0.47	10.79	0.000
	Linear	0.48	0.06	7.95	0.000
Peer Problems					
1	Intercept	0.82	0.32	2.60	0.009
	Linear	-0.62	0.11	-5.39	0.000
	Quadratic	0.04	0.01	4.67	0.000
2	Intercept	0.71	0.14	5.15	0.000
3	Intercept	1.67	0.28	6.03	0.000
	Linear	-0.54	0.11	-4.93	0.000
	Quadratic	0.04	0.01	4.11	0.000
4	Intercept	1.87	0.13	14.97	0.000
5	Intercept	1.04	0.21	4.89	0.000
6	Intercept	1.27	0.36	3.51	0.005
	Linear	0.39	0.09	4.47	0.000

Group 1 is non-engagers (n = 1995), Group 2 is low increasing internalising/low stable-decreasing externalising and peer problems (n = 1550), Group 3 is normative (n = 2190), Group 4 is moderate increasing internalising/moderate decreasing-stable externalising and peer problems (n = 973), Group 5 is low increasing internalising/mixed (moderate-decreasing, high-increasing) externalising/stable peer problems (n = 575), and Group 6 is high chronic-increasing multimorbid (n = 224)

As inspection of both the AvePP and OCC revealed high assignment accuracy and subsequently minimal bias in trajectory group assignment, particularly for groups 4, 5, and 6 (i.e., high AvePP of 81.4, 81.9 and 89.7, respectively; and high OCC 29.5, 54.4, and 281.7, respectively), coupled with the interest in better understanding etiology of groups presenting with elevated and complex problems, group probabilities were extracted. Bivariate analysis (i.e., chi-square and analysis of variance), were then conducted to better understand individual risk factors associated with group membership. Risk factors that were statistically significant for group membership at the bivariate level were examined at the multivariable level using a multinomial logistic regression. The first three trajectory groups exhibiting low levels of internalising, externalising, and peer problems were then combined to form a reference category. This allowed a more in-depth examination of particular risk factors that distinguished groups presenting with more elevated problems from the non-to-low problems groups. Given the potential interest in risk factors across all groups, an additional analysis was also conducted examining risk factors for all groups as compared to the first ‘non-engagers’ group. This analysis was run in a single step which included covariates directly in the trajectory estimation. Results are largely consistent and can be found in the supplementary material, Table 1. All analyses were performed using Stata v14.0. The term significance is used in lieu of statistical significance hereafter.

## Results

### Group-Based Multi-Trajectories

**Fig. 1:**
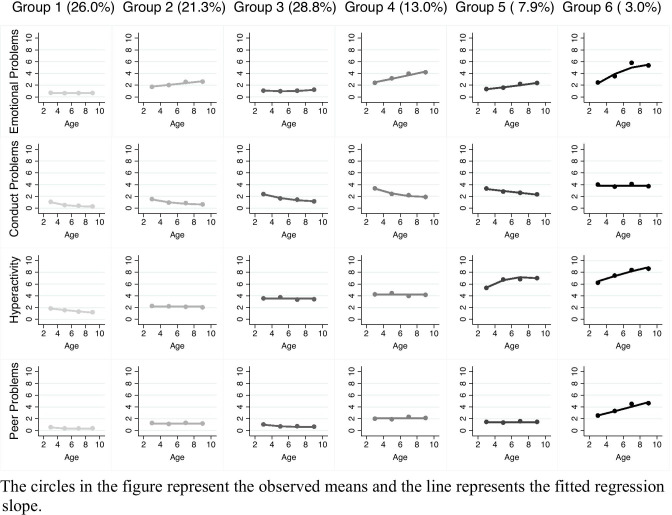
Multi-Trajectories of Internalising, Externalising, and Peer Problems from Early to Late Childhood

Six trajectory groups were identified, presenting with distinguished patterns of concomitant internalising, externalising, and peer problems. The first group was labelled ‘non-engagers’ and included an estimated 26% of the cohort. This group had very low stable internalising problems (i.e., emotional symptoms) and peer problems, and low decreasing externalising problems (i.e., conduct problems and hyperactivity/inattention), from three to nine years of age. The second group included an estimated 21.3% of children and was labelled ‘low increasing internalising/low stable-decreasing externalising and peer problems’. In this group, emotional symptoms were low but increasing over time, whilst peer problems were low and stable. In contrast, conduct problems were low, decreasing from three to seven, and remained stable thereafter. Hyperactivity/inattention was also low but stable. Group 3, the largest group, included an estimated 28.8% of children and was labelled ‘normative’. Similar to group 1, both emotional symptoms and peer problems were low over time, although quadratic in shape. Conduct problems were moderate at age three, decreased to low levels between five and seven, and remained stable from seven to nine years of age. Hyperactivity/inattention bordered a moderate level in this group, although was still within low levels, and remained stable. Group 4, labelled ‘moderate increasing internalising/moderate decreasing-stable externalising and peer problems’, included an estimated 13% of children. Initial emotional symptoms were low in this group but increased linearly to moderate levels by age five and continued upwards until the age of nine. Peer problems were moderate and stable. Conduct problems followed a quadratic trajectory, similar to groups 2 and 3, starting at moderate levels at age three, decreasing until seven and remaining stable between seven to nine years. Hyperactivity/inattention was moderate and stable. Group 5 included an estimated 7.9% of children and was labelled ‘low increasing internalising/mixed (moderate-decreasing, high-increasing) externalising/stable peer problems’. Similar to group 4, emotional symptoms had a linear increasing trajectory over time, albeit at a lower initial level and approaching moderate levels only at age nine. Peer problems were low and stable. Conduct problems followed a linear decreasing trajectory from moderate levels at age three to low levels between five to nine years. In contrast, hyperactivity/inattention was moderate at the age of three, increasing in quadratic form to high levels thereafter. Finally, group 6 consisted of an estimated 3% of children, labelled as ‘high chronic-increasing multimorbid’. This group of children started with moderate to high levels of all internalising, externalising, and peer problems. Emotional symptoms, hyperactivity/inattention and peer problems all increased over time whilst conduct problems remained stable. See Fig. 1 for multi-trajectories and Table 4 for parameter estimates.

### Risk Factors and Group Membership

Chi-square tests and analysis of variance revealed that all risk factors examined at the bivariate level were significant for group membership (see Table 5). For a more comprehensive understanding of the specific risk factors associated with membership in the moderate to elevated groups (i.e., 4, 5, & 6), a multinomial logistic regression was run combining groups 1–3 as the reference category given the normative and low levels of internalising, externalising, and peer problems exhibited in these first three groups (i.e., *x*^*2*^ = 1048.56 (54), *p* =  < 0.001, with Pseudo R^2^ = 0.09). For the moderate increasing internalising/moderate decreasing-stable externalising and peer problems group (group 4), low birth weight was the only significant factor at the individual level, increasing the relative risk ratio for group membership. At the family level, prenatal exposure to smoking, maternal age, maternal education, maternal depression, medical card status and maternal stress were all significant and increased the relative risk ratio. Conversely, a higher quality of attachment was significant and decreased the relative risk ratio. Significant factors increasing the relative risk ratio of membership in the low increasing internalising/mixed (moderate-decreasing, high-increasing) externalising/stable peer problems group (group 5), included child sex, prenatal exposure to smoking, maternal education, single-parent families, social class, and increased maternal stress. Higher quality of attachment was also significant and decreased the relative risk ratio for this group. Finally, significant factors increasing the relative risk ratio for membership in the high chronic-increasing multimorbid group (group 6) included child sex, low birth weight, prenatal exposure to smoking, maternal education, maternal depression, medical card status, and increased maternal stress (see Table 6).

**Table 5:** Bivariate Analysis of Antecedent Risk Factors by Group

	Group 1:	Group 2:	Group 3:	Group 4:	Group 5:	Group 6:	*p*
Individual Factors							
Male	42.6%	42.9%	56.1%	48.9%	68.5%	73.7%	< 0.001
Born preterm	5.1%	6.2%	5.4%	7.5%	6.1%	12.1%	< 0.001
Low birth weight	4.6%	5.2%	4.9%	7.3%	4.2%	13.1%	< 0.001
NICU stay	12.4%	13.9%	13.3%	14.8%	14.1%	22.3%	0.002
Family Factors							
Social Class: Never worked	4.4%	6.0%	7.0%	13.5%	14.6%	17.4%	< 0.001
Single parent	4.2%	7.7%	7.7%	14.8%	17.9%	20.1%	< 0.001
Medical card: Full coverage	14.3%	19.0%	20.0%	30.9%	31.7%	43.3%	< 0.001
Household Smoking	22.3%	28.5%	31.2%	41.2%	37.2%	43.7%	< 0.001
Maternal Factors							
Less than 21 years of age	1.0%	2.2%	3.1%	6.7%	5.7%	5.9%	< 0.001
Primary level/No education	1.0%	1.5%	1.6%	2.6%	1.6%	4.5%	< 0.001
Postnatal depression	1.9%	3.1%	3.2%	7.2%	5.5%	10.7%	< 0.001
Means							
Quality of Attachment	43.2 (2.1)	42.6 (2.5)	42.5 (2.5)	41.8 (2.9)	42.0 (2.7)	41.9 (3.4)	< 0.001
Maternal Stress	29.6 (6.2)	31.8 (6.6)	31.9 (6.3)	34.6 (6.9)	33.6 (6.8)	34.9 (7.6)	< 0.001

Percentages of prevalence displayed for chi-square analysis; means and (standard deviations) presented in analysis of variance. Group 1 is non-engagers, Group 2 is low increasing internalising/low stable-decreasing externalising and peer problems, Group 3 is normative, Group 4 is moderate increasing internalising/moderate decreasing-stable externalising and peer problems, Group 5 is low increasing internalising/mixed (moderate-decreasing, high-increasing) externalising/stable peer problems, and Group 6 is high chronic-increasing multimorbid. Medical card is means-tested and issued on the basis of financial need by health services. There are 2 tiers: “full coverage,” including visits to general practitioners and prescriptions and “general practitioner only,” excluding prescriptions. For maternal education, “primary level/no education” is roughly equivalent to elementary or middle school education in the US; “second level” is roughly equivalent to having graduated high school or having a technical trade/vocational diploma in the US; and “third level” is equivalent to higher education (i.e., college or bachelor’s degree, graduate degree, or doctorate)

**Table 6:** Risk Factors for Group Membership

	Group 4 RRR (SE) [95% CI]	Group 5 RRR (SE) [95% CI]	Group 6 RRR (SE) [95% CI]
Male	1.11(0.08) [0.97–1.27]	2.26(0.21) [1.88–2.71]	3.90(0.58) [2.92–5.22]
Born preterm	0.89(0.16) [0.62–1.26]	0.92(0.22) [0.57–1.48]	0.90(0.26) [0.51–1.60]
Low birth weight	1.48(0.26) [1.06–2.08]	0.72(0.19) [0.43–1.21]	1.95(0.54) [1.13–3.34]
NICU stay	0.97(0.11) [0.77–1.21]	1.02(0.15) [0.77–1.36]	1.40(0.27) [0.96–2.05]
Exposure to household smoking	1.42(0.11) [1.23–1.64]	1.21(0.12) [1.00–1.46]	1.31(0.18) [1.00–1.70]
Maternal age less than 21 years	2.01(0.28) [1.52–2.65]	1.15(0.22) [0.79–1.67]	0.91(0.23) [0.55–1.50]
Maternal education:			
Primary level/no education	1.58(0.29) [1.10–2.27]	0.67(0.21) [0.37–1.22]	3.18(0.90) [1.83–5.53]
Second level	1.30(0.11) [1.10–1.53]	1.44(0.15) [1.17–1.78]	1.88(0.31) [1.35–2.61]
Maternal depression	1.37(0.20) [1.03–1.82]	1.06(0.20) [0.72–1.54]	2.16(0.46) [1.42–3.29]
Medical card status:			
Full coverage	1.28(0.12) [1.06–1.54]	1.14(0.14) [0.90–1.45]	2.42(0.41) [1.74–3.37]
General practitioner only	1.02(0.22) [0.67–1.57]	1.42(0.35) [0.88–2.29]	3.05(0.90) [1.71–5.44]
Single parent	1.25(0.15) [0.10–1.57]	1.75(0.25) [1.32–2.32]	1.31(0.26) [0.88–1.94]
Social class:			
Other non-manual/skilled-manual	1.19(0.11) [0.10–1.41]	1.18(0.14) [0.94–1.48]	1.32(0.23) [0.92–1.85]
Semi-skilled/unskilled manual	1.20(0.16) [0.93–1.55]	1.44(0.24) [1.04–1.99]	0.65(0.18) [0.38–1.12]
All others gainfully occupied and unknown	1.45(0.72) [0.55–3.83]	0.30(0.42) [0.02–4.47]	0.73(0.89) [0.07–8.04]
Never worked/no class	1.22(0.19) [0.89–1.66]	1.97(0.39) [1.34–2.89]	1.63(0.43) [0.97–2.73]
Quality of attachment	0.94(0.01) [0.92–0.97]	0.91(0.02) [0.88–0.94]	0.98(0.03) [0.93–1.03]
Maternal stress	1.06(0.01) [1.05–1.08]	1.03(0.01) [1.01–1.04]	1.06(0.01) [1.04–1.08]

N = 7,145. The comparison trajectory group combined the non-engagers, low increasing internalising/low stable-decreasing externalising and peer problems, and normative trajectories. Group 4 is moderate increasing internalising/moderate decreasing-stable externalising and peer problems, Group 5 is low increasing internalising/mixed (moderate-decreasing, high-increasing) externalising/stable peer problems, and Group 6 is high chronic-increasing multimorbid. The reference category for maternal education was third level. The reference category for medical card status was no medical card, indicative of higher income. The comparison category for social class was professional/managerial. The relative risk ratio is presented along with the (standard error) and the [95% confidence intervals]. A relative risk ratio of < 1 is indicative of a lower likelihood of belonging in the referred trajectory group, whereas > 1 is indicative of higher likelihood in the trajectory group

## Discussion

To the best of my knowledge, this study is the first to have modelled concomitant trajectories of internalising, externalising, and peer problems from early to late childhood, using a group-based multi-trajectory approach, whilst examining antecedent risk factors associated with group membership. A common method for investigating developmental psychopathologies is to examine individuals who deviate from the majority (e.g., Cicchetti & Cohen, 2006; Laursen & Hoff, 2006; Bergman et al., 2006). Whilst this approach is well suited in many instances, it risks potential dilution of unique clusters of individuals, in particular when examining trajectories of homotypic and heterotypic comorbidity, within population-based samples. An advantage of the multi-group-based approach is the identification of numerous clusters of children exhibiting distinct patterns of simultaneous behaviours over time. In using the GUI Infant cohort, the results revealed that a 6-group model best fit the data. Complex and comorbid patterns of internalising, externalising, and peer problems were found in groups presenting with higher problems. Both continuity and change over time were observed within trajectory groups, along with common and specific risk factors predicting group membership. Given the extensively documented maladaptive outcomes associated with internalising, externalising, and peer problems when examined individually (e.g., Bevilacqua et al., 2018; Rivenbark et al., 2018; Fairchild et al., 2019; Gutman & McMaster, 2020; Galera et al., 2021; Orri et al., 2018), identification of comorbid and multimorbid trajectories using a population-based sample, along with a better understanding of antecedent risk factors associated with group membership, sheds several important new insights.

First, within the 6-group model, there was little evidence to support the existence of a ‘pure’ trajectory group in early to late childhood, for internalising, externalising, or peer problems, contrary to the hypothesis and previous findings (Fanti & Henrich, 2010; Patalay et al., 2017). Instead, difficulties in one domain appeared to indicate the presence of difficulty in another domain (both homo- and heterotypic comorbidity). Whilst distinct, groups 1–3 all exhibited low levels of internalising, externalising, and peer problems. That is, 76.1% of the sample fell within a normal range of behaviours, albeit with variation (i.e., increases/decreases/stability) over time. This is a higher proportion of children exhibiting no to low internalising, externalising, and peer problems as compared to the two previous studies modelling joint trajectories of internalising and externalising problems with similar age groups (Fanti & Henrich, 2010; Patalay et al., 2017). However, a notable difference is the inclusion of peer problems modelled in the trajectories within the current study. 

With the exception of hyperactivity/inattention, groups 4 and 5 displayed similar patterns of behaviours across time and comprised a combined 20.9% of the sample. In both groups, emotional symptoms were found to increase linearly over time, although at higher levels for group 4. Whilst conduct problems were quadratic in shape for group 4 and linear for group 5, decreases from moderate levels at age three, to low levels by age nine were observed in both groups, following an expected developmental pattern (Shaw et al., 2003). The experience of peer problems in both groups was moderate but stable across time. In contrast, hyperactivity/inattention for group 4 was moderate but stable across time, whereas for group 5 it was quadratic in shape, starting at moderate levels and increasing before tapering off at high levels thereafter. Whilst the analytic approach can only infer multimorbidity and not directionality between behaviours, it could be hypothesised that the early moderate levels of conduct problems and moderate-high levels of hyperactivity/inattention resulted in the observed stability of moderate peer problems in these groups across time. For instance, notable links have been found to support the early presence of homotypic comorbidity of conduct problems and hyperactivity/inattention with future peer problems in childhood (Andrade & Tannock, 2014; Becker et al., 2012; Gresham et al., 1998), suggestive of successive heterotypic comorbidity. Moreover, stable levels of peer problems may contribute to increasing emotional symptoms over time (Bond et al., 2001; O'Brennan et al., 2009).

Group 6 included an estimated 3% of the sample and presented with chronic-increasing levels of elevated problems across domains. Levels of conduct problems started high and remained stable. In contrast, all other behaviours increased from moderate to high between three and nine years of age. The elevated levels across all behaviours highlight both concurrent and successive homotypic and heterotypic comorbidity in this group, another notable finding of the study. Much attention has been paid to homotypic comorbidity with somewhat less attention focusing on heterotypic comorbidity. This is particularly the case for mapping trajectories of possible concomitant internalising and externalising problems starting in early childhood. The increasing level of emotional symptoms, hyperactivity/inattention, and peer problems against the backdrop of stably elevated conduct problems suggests dependency across behaviours in a small group of children. High comorbidity between conduct problems and hyperactivity/inattention in developmental studies is not uncommon (Angold et al., 1999; Beauchaine et al., 2010). Theoretically, the combined presence of elevated conduct problems and hyperactivity/inattention may result in increased difficulties and rejection by peers over time, cascading into increased emotional symptoms. On the other hand, early emotional symptoms may result in withdrawal from peers and subsequent difficulty with attention during daily tasks. However, this latter hypothesis would not account for the early elevated and stable conduct problems found in this group. The identification of a smaller chronic/elevating group is line with previous studies examining developmental psychopathologies, which in individual and joint trajectories have suggested < 10% of children following a high-chronic developmental course (e.g., Barker et al., 2008; Nagin & Tremblay, 1999; Patalay et al., 2017; Shaw et al., 2003). Given the modelling of concomitant internalising, externalising, and peer problems, the prevalence rate of the chronic group in this study was on the lower end, with approximately 3% of children.

Knowledge of potential common versus specific risk factors associated with group-membership is critical to furthering our understanding of differing patterns of concomitant behavioural problems across childhood. Only three risk factors were uniformly found to predict membership in all elevated groups as compared to the combined reference group. These included prenatal exposure to smoking, maternal education, and maternal stress. There remains debate as to whether the link between prenatal exposure to smoking and consequent behavioural difficulties is direct or an artefact of characteristics associated with mothers who smoke (e.g., Roza et al., 2009). In the current study, prenatal exposure to smoking was measured by the number of household members smoking during pregnancy, which may or may not have included the mothers, rather than maternal engagement alone. It is possible then that this finding may support a direct link between in-utero exposure to toxins and future emotional and behavioural problems. More work in this area is however first needed before conclusions can be drawn. Similarly to previous studies, lower maternal education was also a common predictor (e.g., Nagin & Tremblay, 2001). Children with mothers who had primary/no or second level education as compared to third level were approximately 1.5 times at greater risk for belonging to groups 4 and 5. The relative risk ratio doubled for children in group 6, whereby having primary/no education increased the risk of membership by 3.1 and second level education by 1.8. A higher level of maternal stress was also a common risk factor for group membership in elevated groups. For each point increase in maternal stress, there was a 6%, 2%, and 5% increase in membership in groups 4, 5, and 6, respectively.

Three factors, low birth weight, maternal depression, and medical card status, were commonly associated with increased risk for membership in groups 4 and 6. Notably, the relative risk ratios for all risk factors were consistently larger for membership in group 6. The relative risk ratio for low birth weight in group 6 was almost two-fold as compared to the reference group, possibly driven by the high levels of both internalising problems and hyperactivity/inattention in this group (Aarnoudse-Moens et al., 2009; Nigg & Breslau, 2007). In group 4, the relative risk ratio was 1.4. Maternal depression slightly increased risk of membership in group 4 (RRR: 1.3), but more than doubled the risk of membership in group 6 (RRR: 2.1). This finding was not surprising given the characteristically poorer quality interactions between children and mothers suffering from depression, in addition to increased genetic risk of mental health difficulties (Kim-Cohen et al., 2005). Medical card status, a proxy of income, was also found to increase the risk slightly for group 4, but more substantially for group 6 (RRR: 2.4 and 3.0 for full and partial coverage respectively).

Boys were at a two-fold higher risk for membership in group 5, and almost four-fold the risk for membership in group 6. Whilst boys are routinely found at risk for elevated trajectories of externalising problems (e.g., Girard et al., 2019), the evidence is mixed when examining internalising problems (Dekker et al., 2007; Mesman et al., 2001). These results suggest that heterotypic comorbidity of internalising and externalising problems increases the risk of membership in elevated groups for boys in particular. Of interest, boys were not at higher risk for membership in group 4, which presented similarly to the low increasing internalising/mixed (moderate-decreasing, high-increasing) externalising/stable peer problems group (group 5) on emotional symptoms (albeit at lower levels), conduct problems and peer problems, but not on hyperactivity/inattention. In the latter group, hyperactivity/inattention increased over time whereas in the former group hyperactivity/inattention remained stable. This would suggest that the presence of either increasing difficulties in hyperactivity/inattention, lower levels of emotional symptoms, or both, resulted in this increased risk in group membership for boys. This result is notable in that it suggests that complex and concomitant internalising, externalising, and peer problems, particularly when hyperactivity/inattention is high, is more common amongst boys.

Quality of attachment was a common factor for membership in both groups 4 and 5. More specifically, risk of group membership decreased by 6% and 9%, respectively, for each point increase, suggesting that early positive attachment played a protective role against moderate levels of internalising, externalising, and peer problems, but not for the highest levels of problems.

Three factors were specific to group membership and included young maternal age (group 4), single parents and lower social class (group 5). Perhaps most surprising is young maternal age not increasing the risk of membership in group 6 (Lee et al., 2020). Given the low number of mothers under 21 years of age in this sample, replication is however needed. Taken together, this study suggests some overlap of antecedent risk factors common to a couple of the elevated groups, although only three factors uniformly increased the risk for all three elevated groups. It would be of great interest to see future studies that extend the current work by examining these trajectories into adolescence, a transition period marked by great challenges for many youngsters. Moreover, given that the current study focuses uniquely on predictors of group membership at 9 months, future work would do well to build upon these findings by examining time-varying predictors (e.g., cognitive abilities, academic outcomes, parenting practices), which may provide additional understanding around continuity and change within trajectory groups, along with informing protective and risk factors across development.

Despite the noteworthy strengths of this study, including being the first to use a person-centered approach in modelling concomitant trajectories of internalising, externalising, *and* peer problems, from early to late childhood, whilst using a large and representative population-based cohort, consequently allowing unbiased prevalence rates, some limitations need mention. First, parent reports were used to collect information on both risk factors and children’s outcomes. Thus, shared method variance is a possible concern and future studies would be well placed in using multiple informants. Second, attrition across waves resulted in the loss of almost a third of the initial cohort, with higher attrition amongst families at greater social disadvantage. However, to circumvent the disproportionate distribution, sampling weights were used which resulted in a 0.5% difference between the included sample and the population with respect to participant characteristics. Additional group-based multi-trajectory analysis using all participants with data on the SDQ for at least one time (n = 10,170) was also explored given the high attrition rates. Results were largely consistent (i.e., a 6-group model best fit the data, the shape of trajectories remained unchanged), although the proportion of group membership was slightly increased for the elevated groups when using the entire sample. Third, data was only collected up to age nine. Extending trajectories into adolescence would provide additional opportunities to better understand whether groups with elevated problems continue on the same path and whether potential adolescent-onset groups would emerge. Fourth, whilst the SDQ is one of the most widely used behavioural screening measures (Stone et al., 2010), Cronbach’s alpha, which represents the ‘lower bound’ of the true reliability (Cronbach, 1951), was below the desired threshold of ≥ 0.70 for three of the four scales in the current study. A potential consequence is the underestimation of behavioural trajectories and their associated risk factors given that attenuation, via the reduction of maximum observable associations between variables, is more likely (Schmitt, 1996). Fifth, given the interest in understanding risk/protective factors specific to trajectory groups with elevated behavioural problems, the primary analysis was conducted in a two-stage process. That is, trajectory groups were extracted following the modelling of groups to collapse the first three (no-low) trajectory groups, as there is no way to collapse groups within the model itself. Consequently, groups were treated as observed. Finally, whilst risk factors were identified, inference of risk mechanisms could not be asserted given the observational nature of the study. Future work should build on this study by examining risk mechanism.

## Conclusions

Using a person-centered approach with a population-based cohort, 6 distinct subgroups of children presenting with concomitant internalising, externalising, and peer problems was found, providing unbiased prevalence rates (Angold et al., 1999). Both homotypic and heterotypic comorbidity/multimorbidity was found in groups with elevated behavioural problems. Targets for intervention programming were identified, and despite some common overlap in predictors across groups, the combination of predictors specific to each group would suggest tailored programming. For those children with the most acute levels of concomitant internalising, externalising, and peer problems, the current results would suggest targeting families with boys who were born with low birth weight, with mothers who have lower levels of education and fewer financial resources. Components of this programming should include education and resources for combating maternal postnatal depression and stress levels, along with information and support to reduce prenatal exposure to smoking for all family members in the household.

## Supplementary Information

Below is the link to the electronic supplementary material.

Supplementary file1 (DOCX 25 KB)

## Data Availability

The Growing Up in Ireland data that support the findings of this study are available from the Irish Social Science Data Archive, https://www.ucd.ie/issda/.
