# Supplementary material for: Concomitant Trajectories of Internalising, Externalising, and Peer Problems Across Childhood: a Person-centered Approach
Source: Res Child Adolesc Psychopathol. 2021 Jul 19;49(12):1551–65. doi: 10.1007/s10802-021-00851-8 (PMC8557151; doi:10.1007/s10802-021-00851-8)
Supplement: Supplementary file 1 — Supplementary file1 (DOCX 25 KB) [file 10802_2021_851_MOESM1_ESM.docx]

Supplementary Table 1: Risk Factors for Group Membership

| Group |  | Coefficient Estimate | SE | T | *p* |
| --- | --- | --- | --- | --- | --- |
| 1 | Constant | (0.000) | . | . | . |
|  |  |  |  |  |  |
| 2 | Constant | 1.79 | 1.44 | 1.24 | 0.215 |
|  | Child sex (boy): | -0.08 | 0.15 | -0.50 | 0.616 |
|  | Born preterm (yes): | 0.41 | 0.30 | 1.39 | 0.165 |
|  | Low birth weight (yes): | -0.53 | 0.33 | -1.59 | 0.112 |
|  | NICU stay (yes): | 0.23 | 0.18 | 1.28 | 0.201 |
|  | Household Smoking (yes): | 0.35 | 0.13 | 2.63 | 0.009 |
|  | Maternal age (less than 21 years of age): | 0.14 | 0.55 | 0.26 | 0.799 |
|  | Lower maternal education: | 0.25 | 0.15 | 1.65 | 0.099 |
|  | Maternal depression (yes): | 0.28 | 0.36 | 0.77 | 0.440 |
|  | Medical card coverage (yes): | 0.22 | 0.21 | 1.04 | 0.299 |
|  | Single parent (yes): | 0.90 | 0.32 | 2.85 | 0.004 |
|  | Social Class (semi-skilled, unskilled, never worked): | -0.10 | 0.23 | -0.43 | 0.670 |
|  | Quality of attachment: | -0.10 | 0.03 | -3.20 | 0.001 |
|  | Maternal stress: | 0.07 | 0.01 | 6.23 | 0.000 |
|  |  |  |  |  |  |
| 3 | Constant | 3.46 | 1.22 | 2.84 | 0.005 |
|  | Child sex (boy): | 0.62 | 0.12 | 5.40 | 0.000 |
|  | Born preterm (yes): | 0.13 | 0.30 | 0.45 | 0.654 |
|  | Low birth weight (yes): | -0.27 | 0.31 | -0.87 | 0.383 |
|  | NICU stay (yes): | 0.14 | 0.16 | 0.90 | 0.370 |
|  | Household Smoking (yes): | 0.42 | 0.12 | 3.54 | 0.000 |
|  | Maternal age (less than 21 years of age): | 0.71 | 0.47 | 1.51 | 0.130 |
|  | Lower maternal education: | 0.36 | 0.12 | 3.08 | 0.002 |
|  | Maternal depression (yes): | -0.08 | 0.37 | -0.20 | 0.841 |
|  | Medical card coverage (yes): | 0.18 | 0.16 | 1.10 | 0.270 |
|  | Single parent (yes): | 0.54 | 0.30 | 1.79 | 0.074 |
|  | Social Class (semi-skilled, unskilled, never worked): | -0.13 | 0.19 | -0.69 | 0.488 |
|  | Quality of attachment: | -0.14 | 0.03 | -5.37 | 0.000 |
|  | Maternal stress: | 0.06 | 0.01 | 5.95 | 0.000 |
|  |  |  |  |  |  |
| 4 | Constant | 1.10 | 1.55 | 0.71 | 0.479 |
|  | Child sex (boy): | 0.26 | 0.15 | 1.72 | 0.086 |
|  | Born preterm (yes): | -0.07 | 0.39 | -0.18 | 0.860 |
|  | Low birth weight (yes): | 0.37 | 0.38 | 0.98 | 0.329 |
|  | NICU stay (yes): | -0.09 | 0.22 | -0.41 | 0.683 |
|  | Household Smoking (yes): | 0.72 | 0.15 | 4.71 | 0.000 |
|  | Maternal age (less than 21 years of age): | 1.41 | 0.48 | 2.93 | 0.003 |
|  | Lower maternal education: | 0.57 | 0.15 | 3.85 | 0.000 |
|  | Maternal depression (yes): | 0.41 | 0.36 | 1.13 | 0.258 |
|  | Medical card coverage (yes): | 0.41 | 0.19 | 2.12 | 0.034 |
|  | Single parent (yes): | 0.86 | 0.31 | 2.79 | 0.005 |
|  | Social Class (semi-skilled, unskilled, never worked): | 0.06 | 0.24 | 0.26 | 0.793 |
|  | Quality of attachment: | -0.16 | 0.03 | -5.07 | 0.000 |
|  | Maternal stress: | 0.13 | 0.01 | 10.10 | 0.000 |
|  |  |  |  |  |  |
| 5 | Constant | 3.71 | 1.83 | 2.02 | 0.043 |
|  | Child sex (boy): | 1.22 | 0.18 | 6.76 | 0.000 |
|  | Born preterm (yes): | 0.40 | 0.38 | 1.07 | 0.284 |
|  | Low birth weight (yes): | -0.63 | 0.46 | -1.36 | 0.175 |
|  | NICU stay (yes): | 0.13 | 0.23 | 0.54 | 0.586 |
|  | Household Smoking (yes): | 0.50 | 0.17 | 2.90 | 0.004 |
|  | Maternal age (less than 21 years of age): | 0.36 | 0.55 | 0.65 | 0.514 |
|  | Lower maternal education: | 0.55 | 0.17 | 3.35 | 0.001 |
|  | Maternal depression (yes): | 0.46 | 0.41 | 1.10 | 0.269 |
|  | Medical card coverage (yes): | 0.41 | 0.22 | 1.87 | 0.061 |
|  | Single parent (yes): | 1.20 | 0.34 | 3.52 | 0.000 |
|  | Social Class (semi-skilled, unskilled, never worked): | 0.27 | 0.24 | 1.10 | 0.272 |
|  | Quality of attachment: | -0.20 | 0.04 | -5.63 | 0.000 |
|  | Maternal stress: | 0.08 | 0.01 | 5.55 | 0.000 |
|  |  |  |  |  |  |
| 6 | Constant | -3.20 | 3.31 | -0.97 | 0.334 |
|  | Child sex (boy): | 1.79 | 0.27 | 6.53 | 0.000 |
|  | Born preterm (yes): | 0.25 | 0.48 | 0.51 | 0.609 |
|  | Low birth weight (yes): | 0.21 | 0.52 | 0.39 | 0.694 |
|  | NICU stay (yes): | 0.49 | 0.29 | 1.68 | 0.092 |
|  | Household Smoking (yes): | 0.65 | 0.22 | 2.95 | 0.003 |
|  | Maternal age (less than 21 years of age): | 0.26 | 0.60 | 0.44 | 0.664 |
|  | Lower maternal education: | 1.12 | 0.24 | 4.61 | 0.000 |
|  | Maternal depression (yes): | 0.90 | 0.49 | 1.83 | 0.068 |
|  | Medical card coverage (yes): | 1.26 | 0.27 | 4.61 | 0.000 |
|  | Single parent (yes): | 1.21 | 0.40 | 3.02 | 0.003 |
|  | Social Class (semi-skilled, unskilled, never worked): | -0.16 | 0.27 | -0.57 | 0.566 |
|  | Quality of attachment: | -0.11 | 0.07 | -1.67 | 0.096 |
|  | Maternal stress: | 0.10 | 0.02 | 5.94 | 0.000 |

Note: Group 1, the non-engagers group, is the comparison group.
